# Age-related differences in risk factors, clinical characteristics, and outcomes for intracerebral hemorrhage

**DOI:** 10.3389/fnagi.2023.1264124

**Published:** 2023-11-03

**Authors:** Chu Chen, Yanfang Xie, Mingjun Pu, Lan Deng, Zuoqiao Li, Tiannan Yang, Hao Yin, Zhehao Zhang, Xinni Lv, Xueyun Liu, Jing Cheng, Qi Li

**Affiliations:** ^1^Department of Neurology, First Affiliated Hospital of Chongqing Medical University, Chongqing, China; ^2^Department of Neurology, Second Affiliated Hospital of Anhui Medical University, Anhui, China; ^3^Department of Neurology, Third Affiliated Hospital of Chongqing Medical University, Chongqing, China

**Keywords:** intracerebral hemorrhage, outcome, predictor, risk factor, young patients

## Abstract

**Background and purpose:**

Intracerebral hemorrhage (ICH) is a severe form of stroke that remains understudied in the young adults. We aimed to investigate the clinical presentation, and risk factors associated with ICH in this age group and compare them to older patients.

**Methods:**

Our study included ICH patients admitted between March 2016 and December 2021 in the First Affiliated Hospital of Chongqing Medical University from our ongoing prospective cohort database. Demographic characteristics, etiology, risk factors, and clinical outcomes were compared between elderly and young patients. Furthermore, logistic regression analysis was employed to explore risk factors associated with the functional outcome at 3-months.

**Results:**

We selected 1,003 patients (mean age, 59.9 ±13.8 years old), 746 (74.4%) patients were aged >50 years. The logistic regression analysis showed young patients have a higher proportion of secondary ICH, higher white blood cell count and higher body mass index (BMI), but less diabetes mellitus. Of all patients, predictors of 3-month functional independence was first-ever ICH and age ≤50 years. The history of nephropathy and stroke, higher baseline NIHSS score, larger hematoma volume, and the presence of hydrocephalus were associated with poor outcomes. And the white blood cell count could significantly influence the prognosis among young ICH patients. Three-month functional outcome based on modified Rankin scale score was better in young patients than the elderly (OR, 1.232; 95% CI, 1.095–1.388; *p* < 0.001).

**Conclusions:**

The highest incidence of ICH occurs in the age groups of 50–59 and 60–69. ICH in young adults had higher white blood cell and BMI compared to the elderly, and differs in etiological distribution. The young patients also had similar short-term mortality but more favorable functional outcomes than the elderly. Furthermore, NIHSS score and larger hematoma volumes were associated with poor outcome in all patients.

## Introduction

Spontaneous intracerebral hemorrhage (ICH) is a devastating form of stroke that could results in focal neurological deficits and high mortality rates, which accounts for 10–20% of all strokes (van Asch et al., 2010). Although ICH traditionally perceived as a disease of older age, the prevalence in younger individuals is increasingly emerging as a public health issue (Krishnamurthi et al., 2014), often leading to permanent disability and imposing a heavy burden on society and economy.

The incidence of ICH in Asian populations was observed approximately double that in Western countries (Jacobs et al., 2002; van Asch et al., 2010), indicating a significant ethnic disparity in the occurrence. Considering the relatively longer life span of young patients, it is critical to clarify the risk factors affecting their functional outcome. However, few studies have investigated the risk factor profile and underlying etiologies for ICH among Chinese young patients (Qureshi et al., 2001, 2009; Keep et al., 2012). Therefore, there is a critical need for studies that targeting young adults to improve our understanding of the impact and develop appropriate prevention and treatment strategies.

In this study, we aimed to investigate the differences in risk factors and clinical characteristics in young and old patients, and to analyze the factors that influence early mortality and functional outcomes between different age groups.

## Methods

### Patients and study design

We included ICH patients treated at the First Affiliated Hospital of Chongqing Medical University between March 2016 and December 2021, using data from our ongoing prospective cohort database. The study was conducted in accordance with local ethical framework, and informed consent was obtained from all participants or their legal surrogates.

The inclusion criteria were as follows: (1) age ≥18 years old, (2) diagnosed with non-traumatic ICH, and (3) undergone a CT scan within 24 h after the onset of symptoms. Patients with ICH due to primary intraventricular hemorrhage, tumor bleeding, hemorrhagic transformation of a cerebral infarction, or missing medical records were excluded.

### Assessment of clinical characteristics

This study assessed risk factors and symptoms in the study patients, including Glasgow Coma Scale (GCS) and National Institutes of Health Stroke Scale (NIHSS) scores, which were recorded by trained neurologist upon arrival. The location of hematoma was identified on baseline CT scans and classified into different regions, including basal ganglia, thalamus, lobar, brainstem, and cerebellum. We calculated hematoma volumes using the ABC/2 method (where A is the greatest hemorrhage diameter by CT, B is the diameter 90° to A, and C is the approximate number of CT slices with hemorrhage multiplied by the slice thickness) (Kothari et al., 1996). Moreover, we recorded the medical history of neurovascular risk factors for ICH, such as hypertension, diabetes mellitus, dyslipidemia, and body mass index (BMI).

Furthermore, we classified the etiology of ICH based on patients' medical history and further brain imaging findings (computer tomographic angiography, magnetic resonance angiography) of each patient. Primary ICH is defined as caused by spontaneous rupture of small blood vessels in the brain, which is often associated with hypertension or cerebral amyloid angiopathy. Secondary ICH is mainly caused by underlying conditions such as intracranial cavernous hemangioma, arteriovenous malformations, arterial aneurysm, anticoagulant associated ICH, moyamoya vasculopathy (Cordonnier et al., 2018). Based on previous research, a young adult patient with ICH is typically defined as an individual between the age 18 and 50 (Tatlisumak et al., 2018).

Hypertension was defined as systolic blood pressure (SBP) ≥140 mmHg and/or diastolic blood pressure (DBP) ≥90 mmHg with repeated examination, or if they were or had been on antihypertensive medication at any time before onset (Unger et al., 2020). Diabetes mellitus was defined as self-reported history, or baseline fasting serum glucose ≥ 7.0 mmol/L (≥ 126 mg/dl), non-fasting serum glucose ≥ 11.1 mmol/L (≥ 200 mg/dl), glycated hemoglobin (HbA1C) ≥ 48 mmol/mol (≥ 6.5% by the Diabetes Control and Complications Trial) or the use of glucose-lowering drugs before onset (American Diabetes Association, 2010). Dyslipidemia was diagnosed if the fasting blood lipid test fulfilling at least one of the following criteria: total cholesterol (TC) ≥6.22 mmol/L, triglyceride (TG) ≥2.26 mmol/L, low-density lipoprotein-cholesterol (LDL-c) ≥4.14 mmol/L, high-density lipoprotein-cholesterol (HDL-c) <1.04 mmol/L, self-reported history, or long-term use of lipid-lowering agents (Stone et al., 2014). Chronic kidney disease (CKD) was ascertained from in-person interviews or baseline estimated glomerular filtration rate (eGFR). Patients were diagnosed as having CKD if they had an eGFR < 60 ml/min/1.73 m^2^ for ≥ 3 months (Levey et al., 2005). Patients with liver disease were defined as patients who had been diagnosed with chronic hepatitis or liver cirrhosis, or who showed abnormal laboratory data for aspartate aminotransferase (> 50 IU/l), alanine aminotransferase (> 50 IU/l), or gamma-glutamyl transferase (> 60 IU/l). Body mass index (BMI) was calculated based on measured body height and weight in the medical record.

### Assessment of outcomes

The clinical outcome was measured using the modified Rankin Scale (mRS) score at 3-month, performed by phone interviews or outpatient clinic visits. Favorable outcomes were defined as mRS of 0–2. Case fatality was defined as death occurring within 30 days after the index stroke.

### Statistical analysis

For baseline clinical characteristics, categorical variables were presented as percentages (%) and continuous variables were as mean ± standard deviation (SD) or median and interquartile range (IQR). Chi-square test, Student's *t*-test, or Mann–Whitney *U-*test were used to compare variables between the young and old groups as appropriate. Binary logistic regression analysis with the backward stepwise method was performed to determine the differences between the groups. Variables with a *p* < 0.1 were selected and entered into the logistic regression analysis, and odds ratios (ORs) and 95% confidence intervals (CIs) were calculated. A two-tailed *p* < 0.05 was considered significant. Statistical analysis was conducted using SPSS software version 25.0 (IBM Corp, Armonk, NY).

## Results

### Baseline characteristics

**Figure 1:**
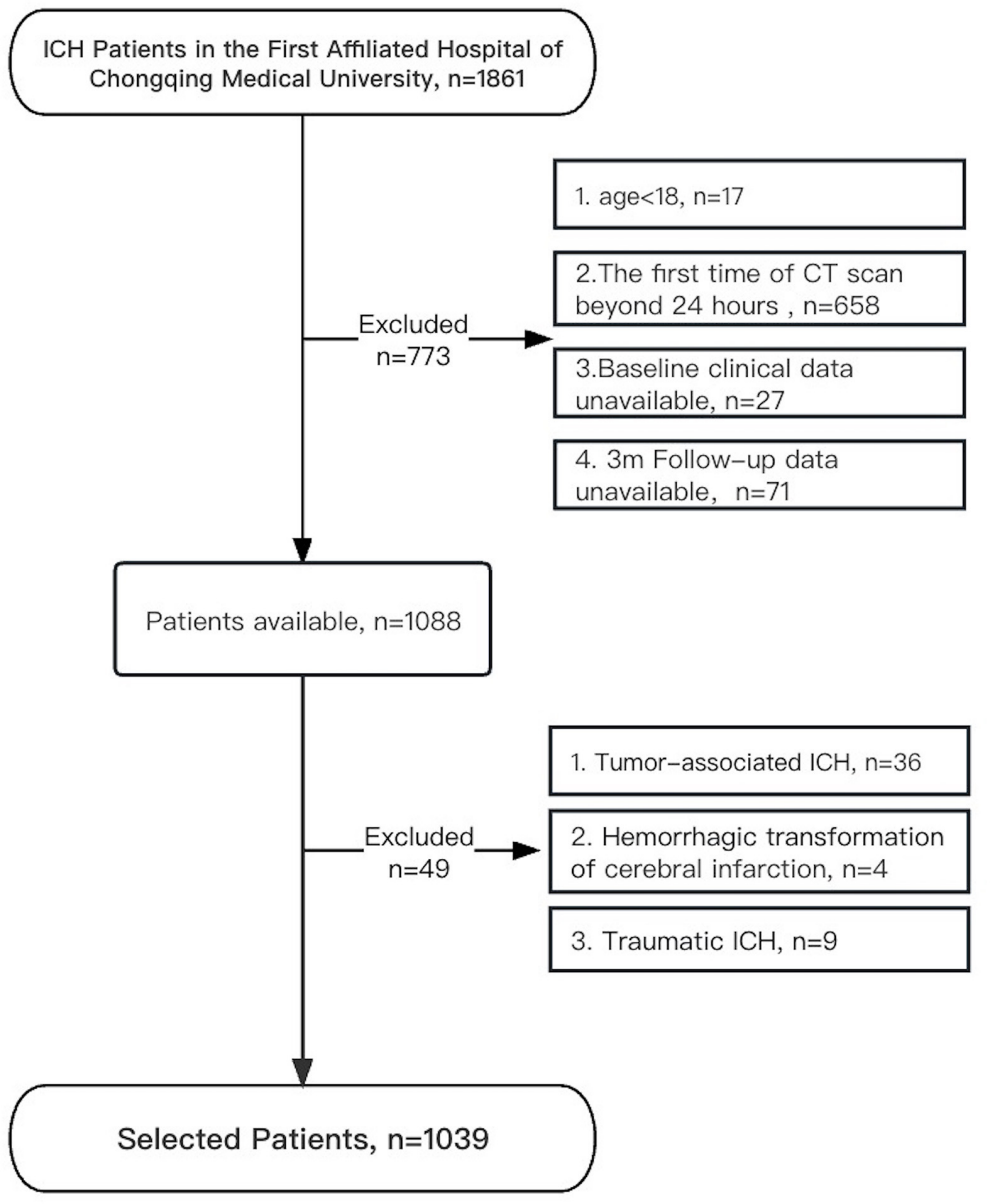
Flowchart for patient selection.

**Figure 2:**
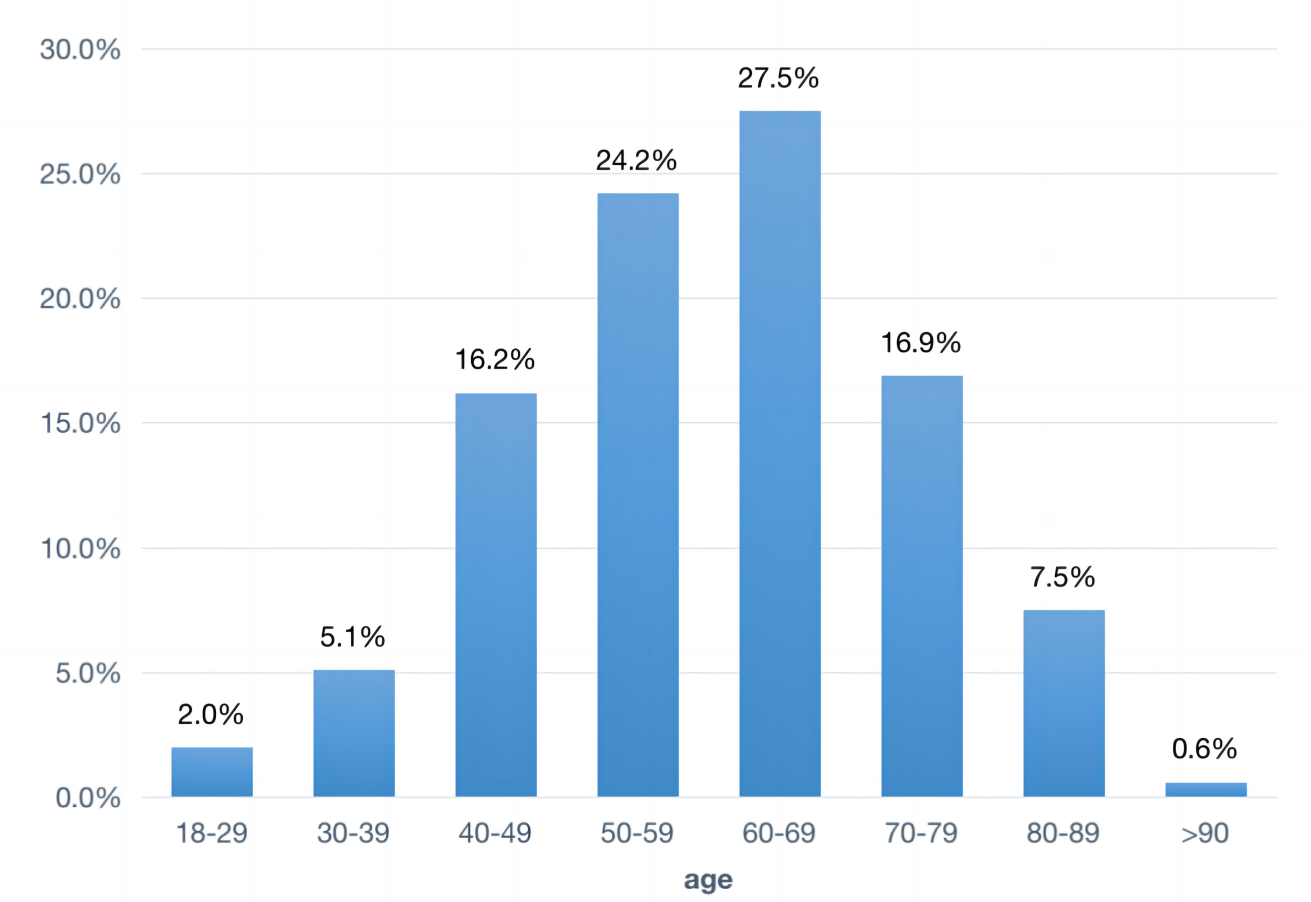
Frequency of ICH in each age group in the study.

During the study period, a total of 1,861 patients with ICH were admitted to our medical center. The patient selection flowchart was illustrated in Figure 1, and 1,003 patients who met the inclusion criteria were included in the final analysis. The mean age of the patients was 59.9 ± 13.8 years, and 68.4% of them (*n* = 686) were male (Table 1). The most frequent location of hematoma was in the basal ganglia (47.7%, *n* = 478), with a median volume of 12.1 (IQR, 4.9–28.7) ml. The majority of cases (51.7%) were between 50–59 and 60–69 years old (Figure 2).

**Table 1:** Comparison of baseline clinical characteristics between young and elderly patients.

**Variables**	**All patients** ***N =* 1003**	** ≤ 50 years** ***N =* 257 (25.6%)**	**>50 years** ***N =* 746 (74.4%)**	***P-*value**
**Demographic**				
Sex, male, *n* (%)	686 (68.4%)	192 (74.7%)	494 (66.2%)	**0.012**
**History of vascular risk factors**				
Hypertension, *n* (%)	704 (70.8%)	158 (62.7%)	546 (73.5%)	**0.001**
Diabetes Mellitus, *n* (%)	178 (18.1%)	33 (13.1%)	145 (19.8%)	**0.018**
Dyslipidemia, *n* (%)	33 (3.3%)	6 (2.3%)	27 (3.6%)	0.319
Chronic kidney disease, *n* (%)	97 (9.7%)	31 (12.1%)	66 (8.8%)	0.133
Liver disease, *n* (%))	49 (4.9%)	13 (5.1%)	36 (4.8%)	0.881
Body mass index, median (IQR)	24.2 (22.0, 26.4)	24.5 (22.5, 27.1)	24.1 (21.6, 26.1)	**0.017**
**Clinical characteristics**				
First-ever ICH, *n* (%)	910 (92.1%)	241 (94.9%)	669 (91.1%)	**0.057**
Previous stroke, *n* (%)	166 (16.8%)	27 (10.6%)	139 (18.9%)	**0.002**
Etiology				**< 0.001**
Primary ICH, *n* (%)	920 (91.7%)	215 (83.7%)	705 (94.5%)	
Secondary ICH, *n* (%)	83 (8.3%)	42 (16.3%)	41 (5.5%)	
Intracranial cavernous hemangioma	9 (0.9%)	2 (0.8%)	7 (0.9%)	
Arteriovenous malformations	33 (3.3%)	24 (9.3%)	9 (1.2%)	
Arterial aneurysm	7 (0.7%)	3 (1.2%)	4 (0.5%)	
Anticoagulant associated ICH	6 (0.6%)	1 (0.4%)	5 (0.7%)	
Moyamoya vasculopathy	11 (1.1%)	6 (2.3%)	5 (0.7%)	
Others	17 (1.7%)	6 (2.3%)	11 (1.5%)	
Systolic BP at onset ≥ 180, *n* (%)	368 (37.3%)	105 (41.3%)	263 (35.9%)	0.121
Baseline GCS, *n* (%)				**0.032**
Median (IQR)	14 (9, 15)	13 (9, 15)	14 (10, 15)	
12-15	672 (67.0%)	156 (60.7%)	516 (69.2%)	
8-11	140 (14.0%)	46 (17.9%)	94 (12.6%)	
0-7	191 (19.0%)	55 (21.4%)	136 (18.2%)	
Baseline NIHSS score, *n* (%)				0.471
Median (IQR)	10 (4, 19)	11 (4, 22)	10 (4, 18)	
0-6	374 (37.3%)	95 (37.0%)	279 (37.4%)	
7-14	286 (28.5%)	67 (26.1%)	219 (29.4%)	
15-42	343 (34.2%)	95 (37.0%)	248 (33.2%)	
Hematoma Location, *n* (%)				**< 0.001**
Basal ganglia	478 (47.7%)	141 (54.9%)	337 (45.2%)	
Thalamus	165 (16.5%)	20 (7.8%)	145 (19.4%)	
Lobar	219 (21.8%)	58 (22.6%)	161 (21.6%)	
Infratentorial lesion	141 (14.1%)	38 (14.7%)	103 (13.8%)	
Hematoma Volume, mL, median (IQR)	12.1 (4.9, 28.7)	15.1 (7.1, 35.5)	11.3 (4.5, 26.3)	**< 0.001**
Baseline Midline shift, *n* (%)	280 (27.9%)	84 (32.7%)	196 (26.3%)	**0.051**
Hydrocephalus, *n* (%)	187 (18.7%)	43 (16.7%)	144 (19.3%)	0.357
White blood cell count, 10^9^/L, median (IQR)	9.5 (7.2, 12.3)	11.1 (8.6, 14.6)	8.9 (6.8, 11.3)	**< 0.001**
Platelet count, 10^9^/L, median (IQR)	181.0 (141.0, 220.0)	197.0 (162.5, 240.0)	175.0 (133.0, 213.0)	**< 0.001**
**Outcomes**			
mRS at discharge, median (IQR)	4 (2, 5)	3 (1, 5)	4 (2, 5)	0.203
90-day mRS, median (IQR)	2 (1, 5)	2 (0, 4)	2 (1, 5)	**0.064**
30-day mortality rate, *n* (%)	185 (18.4%)	45 (17.5%)	140 (18.8%)	0.398

The bold values indicate the variable's *P* value < 0.05 and were included in the next step of logistic regression analysis.

### Difference between young and old groups

Of the 1003 patients, 746 (74.4%) patients were aged >50 years. Baseline characteristics stratified by age group (≤50 years vs. >50 years) were illustrated in Table 1. The younger ICH patients were more likely to be male (74.7 vs. 66.2%, *p* = 0.012), had larger baseline hematoma volume (median, 15.1 vs. 11.3, *p* < 0.001). Primary ICH was considerably more frequent among the elderly (94.5 vs. 87.3%, *p* < 0.01), while secondary ICH occurred more frequently among younger patients (16.3 vs. 5.5%), especially the arteriovenous malformations (9.3 vs. 1.2%).

Older patients had a higher prevalence of hypertension (73.5 vs. 62.7%, *p* = 0.001), diabetes mellitus (19.8 vs. 13.1%, *p* = 0.018), and previous stroke (18.9 vs. 10.6%, *p* = 0.002). However, the admission SBP was significantly higher in the younger population. Furthermore, ICH patients in the younger population had higher white blood cell count (median, 11.1 vs. 8.9, *p* < 0.001) and platelet count (median, 197.0 vs. 175.0, *p* < 0.001). The young group also had a higher BMI (24.5 vs. 24, *p* = 0.017) and were more frequently treated with endotracheal intubation (46.8 vs. 33.8%, *p* < 0.001) and admission in the ICU (89.6 vs. 82.1%, *p* = 0.005) compared to the elderly.

After adjustment for confounding factors in the logistic regression (Table 2), diabetes mellitus history (OR, 2.076; 95% CI, 1.130–3.184; *p* = 0.019), secondary ICH (OR, 0.487; 95% CI, 0.238–0.994; *p* = 0.048), WBC (OR, 0.819; 95% CI, 0.772–0.869; *p* < 0.001), BMI (OR, 0.946; 95% CI, 0.899–0.996; *p* = 0.034) and hematoma location (*p* < 0.001) remained statistically different between the two groups. Young patients also had significant better 90-day outcomes than the elderly patients (OR, 1.232; 95% CI, 1.095–1.388; *p* < 0.001).

**Table 2:** Logistic regression comparison on factors between young and elderly patients.

**Variables**	**Odds ratio**	**95% confidence interval**	***P*-value**
History of diabetes mellitus	2.076	1.130-3.814	0.019
Secondary ICH	0.487	0.238-0.994	0.048
White blood cell count	0.819	0.772-0.869	< 0.001
Body mass index	0.946	0.899-0.996	0.034
Hematoma location			0.002
Thalamus vs. Basal ganglia	2.482	1.336-4.611	0.004
Lobar vs. Basal ganglia	2.043	1.161-3.595	0.013
Other vs. Basal ganglia	2.395	1.246-4.603	0.009
90-day mRS	1.232	1.095-1.388	0.001

### Factors associated with clinical outcome among all patients

**Figure 3:**
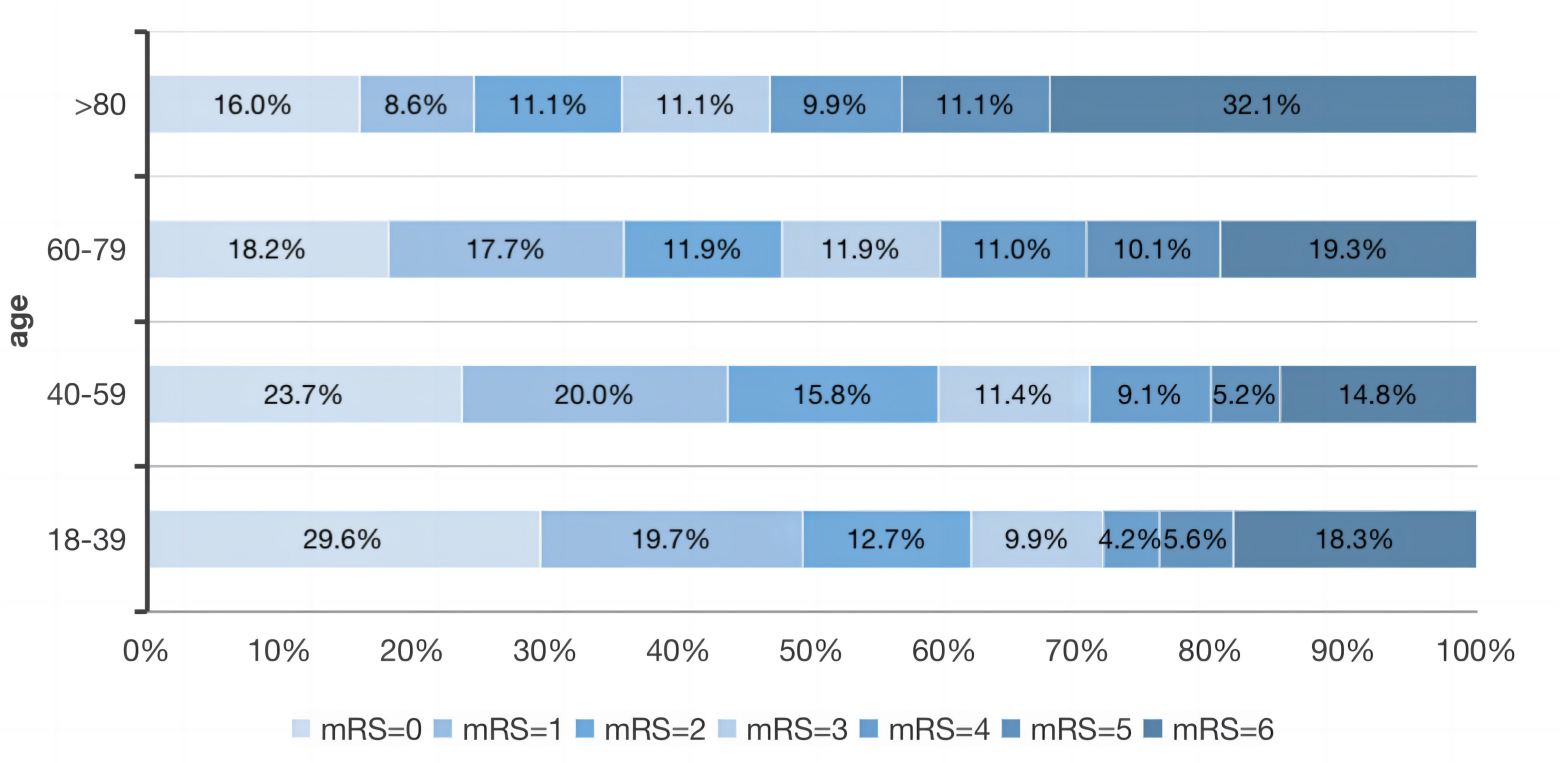
Distribution of modified Rankin Scale scores at the 3-month follow-up for each age group.

Of the 1,003 patients, 527 (52.5%) achieved functional independence (mRS 0–2) at 90-day follow-up (Table 3). Figure 3 showed the mRS scores distributionat the 3-month follow-up for each age group. Logistic regression analysis revealed that age ≤50 years (OR, 0.495; 95% CI, 0.323–0.757; *p* < 0.001) and first-ever ICH (OR, 0.424; 95% CI, 0.181–0.990; *p* = 0.047) decreased the likelihood of poor outcomes. However, the history of chronic kidney disease (OR, 0.4238; 95% CI, 1.045–4.988; *p* = 0.038), previous stroke (OR, 0.519; 95% CI, 0.286–0.940; *p* < 0.030), baseline NIHSS score (OR, 0.906; 95% CI, 0.876–0.937; *p* < 0.001), hematoma volume (OR, 0.965; 95% CI, 0.954–0.976; *p* < 0.001), and hydrocephalus (OR, 0.520; 95% CI, 0.319–0.874; *p* = 0.009) were associated with an increased risk of poor outcomes.

**Table 3:** Univariable and logistic regression comparison on factors associated with 3-month functional independence after ICH of the study cohort.

	**Univariable**			**Logistic**	
	**mRS 0-2** ***N** =* **527 (52.5%)**	**mRS 3-6** ***N** =* **476 (47.5%)**	***P*** **-value**	**OR (95%CI)**	***P-*** **value**
**Demographic**, ***n*** **(%)**			
Age ≤ 50y, *n* (%)	148 (28.1%)	109 (22.9%)	**0.060**	0.495 (0.323-0.757)	0.001
Sex, male, *n* (%)	370 (70.2%)	316 (66.4%)	0.194		
**History of vascular risk factors**					
Hypertension, *n* (%)	363 (69.1%)	341 (72.6%)	0.238		
Diabetes Mellitus, *n* (%)	80 (15.4%)	98 (21.1%)	**0.020**		
Dyslipidemia, *n* (%)	19 (3.6%)	14 (2.9%)	0.556		
Chronic kidney disease, *n* (%)			0.396		
Liver disease, *n* (%)			**0.010**	2.238 (1.045-4.988)	0.038
Body mass index, median (IQR)	24.4 (22.1, 26.7)	24.1 (21.5, 26.1)	0.247		
**Clinical characteristics**					
First-ever ICH, *n* (%)	499 (95.6%)	411 (88.2%)	**< 0.001**	0.424 (0.181-0.990)	0.047
Previous stroke, *n* (%)	61 (11.7%)	105 (22.5%)	**< 0.001**	1.927 (1.064-3.492)	0.030
Primary ICH, *n* (%)	473 (89.8%)	447 (93.9%)	**0.017**		
Systolic BP at onset ≥ 180, *n* (%)	173 (33.1%)	195 (41.9%)	**0.004**		
Baseline GCS, median (IQR)	15 (14, 15)	10 (6, 13)	**< 0.001**		
Baseline NIHSS score, median (IQR)	5 (2, 9)	18 (12, 35)	**< 0.001**	1.104 (1.067-1.142)	< 0.001
Hematoma Location, *n* (%)			0.535		
Basal ganglia	250 (47.4%)	228 (47.9%)			
Thalamus	79 (15.0%)	86 (18.1%)			
Lobar	117 (22.2%)	102 (21.4%)			
Other	81 (15.4%)	60 (12.6%)			
Hematoma volume, median (IQR)	7.6 (2.7, 14.0)	22.9 (10.3, 49.1)	**< 0.001**	1.036 (1.025-1.048)	< 0.001
Baseline Midline shift, *n* (%)	63 (12.0%)	217 (45.7)	**< 0.001**		
Hydrocephalus, *n* (%)	50 (9.5%)	137 (28.8%)	**< 0.001**	1.922 (1.180-3.132)	0.009
White blood cell count, 109/L, median (IQR)	8.5 (6.6, 10.6)	10.7 (8.3, 13.7)	**< 0.001**		
Platelet count, 10^9^/L, median (IQR)	183.0 (146.0, 220.0)	175.5 (131.0, 221.0)	**0.026**		

The bold values indicate the variable's *P* value < 0.05 and were included in the next step of logistic regression analysis.

### Factors associated with clinical outcome among young patients

We further subdivided the young population (*n* = 257) into two groups based on outcome at the 90-day follow-up (Table 4). After adjusting for confounding factors in the logistic regression analysis, we found that first-ever ICH (OR, 0.063; 95% CI, 0.011–0.361; *p* = 0.002), baseline NIHSS score (OR, 1.085; 95% CI, 1.048–1.124; *p* < 0.001), hematoma volume (OR, 1.050; 95% CI, 1.028–1.072; *p* < 0.001), and white blood cell count (OR, 1.123; 95% CI, 1.022–1.234; *p* = 0.016) were associated with poor functional outcome in young ICH patients.

**Table 4:** Univariable and logistic regression comparison on factors associated with 3-month functional independence in young ICH patients.

	**Univariable**			**Logistic**	

	**mRS 0-2** ***N** =* **148 (57.6%)**	**mRS 3-6** ***N** =* **109 (42.4%)**	***P*** **-value**	**OR (95%CI)**	***P*** **-value**
**Demographic**					
Mean age at onset (years, SD)	42.1 (7.7)	42.9 (6.6)	0.610		
Sex, male, *n* (%)	116 (78.4%)	76 (69.7%)	0.115		
**History of vascular risk factors**					
Hypertension, *n* (%)	90 (61.6%)	68 (64.2%)	0.685		
Diabetes Mellitus, *n* (%)	16 (11.0%)	17 (16.0%)	0.247		
Dyslipidemia, *n* (%)	5 (3.4%)	1 (0.9%)	0.197		
Chronic kidney disease, *n* (%)	47 (8.9%)	50 (10.5%)	0.396		
Liver disease, *n* (%)	17 (3.2%)	32 (6.7%)	**0.010**		
Body mass index, median (IQR)	24.6 (22.9, 27.5)	24.3 (21.8, 26.7)	0.232		
**Clinical characteristics**					
First-ever ICH, *n* (%)	144 (97.3%)	97 (91.5%)	**0.039**	0.063 (0.011-0.361)	0.002
Previous stroke, *n* (%)	12 (8.1%)	15 (14.2%)	0.123		
Primary ICH, *n* (%)	122 (82.4%)	93 (85.3%)	0.536		
Systolic BP at onset ≥ 180, *n* (%)	55 (37.2%)	50 (47.2%)	0.110		
Baseline GCS, median (IQR)	15 (14, 15)	10 (6, 13)	**< 0.001**		
Baseline NIHSS score, median (IQR)	5 (2, 9)	18 (12, 35)	**< 0.001**	1.085 (1.048-1.124)	< 0.001
Hematoma Location, *n* (%)			0.381		
Basal ganglia	76 (51.4%)	65 (59.6%)			
Thalamus	14 (9.5%)	6 (5.5%)			
Lobar	36 (24.3%)	22 (20.2%)			
Other	22 (14.9%)	16 (14.7%)			
Hematoma Volume, median (IQR)	10.6 (4.6, 18.2)	35.6 (15.1, 59.8)	**< 0.001**	1.050 (1.028-1.072)	< 0.001
Baseline Midline shift, *n* (%)	25 (16.9%)	59 (54.1%)	**< 0.001**		
Hydrocephalus, *n* (%)	17 (11.5%)	26 (23.9%)	**0.009**		
White blood cell count, 10^9^/L, median (IQR)	9.6 (7.7, 12.1)	13.1 (10.7, 17.5)	**< 0.001**	1.123 (1.022-1.234)	0.016
Platelet count, 10^9^/L, median (IQR)	199.0 (161.0, 240.0)	196.0 (164.0, 245.0)	0.824		

The bold values indicate the variable's *P* value < 0.05 and were included in the next step of logistic regression analysis.

## Discussion

Our results provided the clinical characteristics and prognostic factors of non-traumatic ICH in young patients, and compared the results to those of the elderly. The incidence of ICH is considerably affected by factors such as age, gender, and ethnic disparity (van Asch et al., 2010). Over the past few decades, there has been a global decline in the incidence of ischemic strokes. However, the incidence of hemorrhagic strokes has not followed the same trend (Feigin et al., 2009). Furthermore, non-traumatic ICH in young individuals differs in several key aspects from that in older individuals, and there is a lack of attention and comprehensive guidance for physicians regarding the diagnosis and treatment strategies for ICH in young patients.

Analysis of the 2010 Global Burden of Disease data revealed the incidence of cerebral hemorrhage doubled in younger age groups and was concentrated in low- and middle-income countries (Krishnamurthi et al., 2014). A previous report in 2010 highlighted that the incidence of ICH was 1.9 cases per 100, 000 individuals worldwide among those under 45 years of age, with an escalation to tenfold in the 45–54 age group, and a nearly twentyfold rise in the 55–64 age group (van Asch et al., 2010). Our finding was consistent with this, showing that the highest frequency of ICH in our center occurs in the age groups of 50–59 and 60–69.

Among the participants in our analysis, 68.4% were male, and men appeared to have a higher likelihood of experiencing ICH than women across various age groups. This finding is consistent with a 2015 study in Finland (Koivunen et al., 2015a), where the incidence of non-traumatic ICH was notably higher in men (6.2 cases per 100, 000 individuals; 95% CI 5.0–7.3) than in women (4.0 cases per 100, 000 individuals; 95% CI 3.4–4.7). The male predominance in stroke events can potentially be attributed to the higher prevalence but lower control rate of risk factors (Nzwalo et al., 2018; On et al., 2022).

It has been reported that the etiology of ICH in young patients more often involves structural abnormalities and uncommon underlying factors (Koivunen et al., 2015a). The majority of new evidence in the field of ICH in young individuals comes from genetic research, revealing numerous previously unknown genetic loci associated with brain hemorrhage. These studies have the capacity to offer new insights into the mechanisms of ICH in young patients. In this study, 91.7% cases were caused by primary ICH. We noted that younger population was more frequently associated with secondary ICH, such as arteriovenous malformations, arterial aneurysms, or moyamoya vasculopathy, markedly differing from older patients.

We also showed that the risk factors differ between age groups. Nevertheless, hypertension emerged as the predominant risk factor within both age categories. Younger patients with ICH manifested less frequent diabetes rates but higher body mass index (BMI) compared to older individuals. Prior investigations have established a correlation between elevated BMI, increased high-density lipoprotein (HDL) cholesterol, and the incidence of ICH (Chen et al., 2017; Yang et al., 2020). Therefore, it is necessary to control the aforementioned modifiable risk factors, such as hypertension, diabetes and BMI, to reduce the hemorrhagic stroke occurrence.

Furthermore, our results indicated the white blood cell count was independently associated with poor outcomes in young patients. On the progress of intracerebral hemorrhage, an inflammatory response occurs as a consequence of cerebral vascular bleeding and acute stress reaction, which is accompanied by an elevation in inflammatory markers such as WBC (Di Napoli et al., 2011). In addition, severely ICH patients may experience impaired consciousness and swallowing dysfunction, increasing the risk of developing aspiration pneumonia (Wang et al., 2021). These acute infections can also lead to an elevation in WBC count. Prior research suggested a significant association between elevated inflammatory markers and hematoma growth (Di Napoli et al., 2014; Morotti et al., 2016), as well as a higher incidence of unfavorable clinical outcomes (He et al., 2023).

Prior studies reported that increasing age, baseline NIHSS, baseline GCS, hematoma volume, the presence of hydrocephalus, herniation, renal or heart disease, and multiple hemorrhages were associated with the case-fatality in ICH patients (Hemphill et al., 2001; van Asch et al., 2010; Koivunen et al., 2014, 2015b; Rutten-Jacobs et al., 2014; Yang et al., 2020). Our study were also consistent with these studies, we found the factor associated with patients' 3-month functional independence were the previous medical history of nephropathy and stroke events, age > 50 years, higher NIHSS scores, larger hematoma volumes and the presence of hydrocephalus.

The severity of neurological impairment upon admission demonstrated no significant age-related differences. Similarly, there was no statistical difference between the two groups in 1-month mortality rate (17.5 and 18.8% for young and elderly patients, respectively). However, a marked improvement in outcomes at 90 days was significantly evident among younger ICH patients, suggesting that the rehabilitation rate is more favorable among younger survivors. As evidenced by our study, potential explanations for the observed improved outcomes in younger patients could be attributed to their enhanced neuroplasticity and a lower likelihood of experiencing complications (Koivunen et al., 2014; Yang et al., 2020). Furthermore, the prevalence of vascular risk factors increased with age, younger patients typically exhibit fewer individual risk factors, which contribute to their more favorable outcomes. Emerging evidence supported an aggressive therapeutic approach for spontaneous ICH in young patients, including aggressive blood pressure reduction (Anderson et al., 2013; Qureshi et al., 2016), selective surgical hematoma evacuation (Lai et al., 2005; Koivunen et al., 2014), and intensive rehabilitation.

We recognized several limitations in this study. First, this is a single-center study, our results need to be confirmed by multi-center study. Second, there are additional important young-adult-specific risk factors, including drug abuse, pregnancy, and the postpartum period, however, our current research was lacking in data pertaining to these factors. Third, different levels of nursing care quality may also contribute to the prognosis of ICH patients. It's important to acknowledge that there are variations in the nursing care quality among the patients in this study.

## Conclusion

In summary, our study demonstrates that spontaneous ICH in young adults had higher white blood cell and BMI compared to the elderly, and differs in etiological distribution. The young patients also had similar short-term mortality but more favorable functional outcomes. Furthermore, NIHSS score and larger hematoma volumes were associated with poor outcome in all age groups.

## Data availability statement

The raw data supporting the conclusions of this article will be made available by the authors, without undue reservation.

## Ethics statement

The studies involving humans were approved by the Ethics Committee of the First Affiliated Hospital of Chongqing Medical University. The studies were conducted in accordance with the local legislation and institutional requirements. The human samples used in this study were acquired from a by- product of routine care or industry. Written informed consent was obtained from all participants or their legal surrogates.

## Author contributions

CC: Methodology, Writing—original draft. YX: Methodology, Resources, Writing—original draft. MP: Resources, Writing—review & editing. LD: Resources, Writing—review & editing. ZL: Resources, Writing—review & editing. TY: Resources, Writing—review & editing. HY: Resources, Writing—review & editing. ZZ: Resources, Writing—review & editing. XLv: Resources, Writing—review & editing. XLi: Resources, Writing—review & editing. JC: Resources, Writing—review & editing. QL: Conceptualization, Data curation, Funding acquisition, Supervision, Writing—review & editing.
